# Generating predicate suggestions based on the space of plans: an example of planning with preferences

**DOI:** 10.1007/s11257-022-09327-w

**Published:** 2022-05-31

**Authors:** Gerard Canal, Carme Torras, Guillem Alenyà

**Affiliations:** 1 Department of Informatics, King’s College London, London, United Kingdom; 2 Institut de Robòtica i Informàtica Industrial, CSIC-UPC, Llorens i Artigas 4-6, 08028 Barcelona, Spain

**Keywords:** Planning suggestions, Preference-based planning, Space of plans tree search

## Abstract

Task planning in human–robot environments tends to be particularly complex as it involves additional uncertainty introduced by the human user. Several plans, entailing few or various differences, can be obtained to solve the same given task. To choose among them, the usual least-cost plan criteria is not necessarily the best option, because here, human constraints and preferences come into play. Knowing these user preferences is very valuable to select an appropriate plan, but the preference values are usually hard to obtain. In this context, we propose the Space-of-Plans-based Suggestions (SoPS) algorithms that can provide suggestions for some planning predicates, which are used to define the state of the environment in a task planning problem where actions modify the predicates. We denote these predicates as *suggestible predicates*, of which user preferences are a particular case. The first algorithm is able to analyze the potential effect of the unknown predicates and provide suggestions to values for these unknown predicates that may produce better plans. The second algorithm is able to suggest changes to already known values that potentially improve the obtained reward. The proposed approach utilizes a Space of Plans Tree structure to represent a subset of the space of plans. The tree is traversed to find the predicates and the values that would most increase the reward, and output them as a suggestion to the user. Our evaluation in three preference-based assistive robotics domains shows how the proposed algorithms can improve task performance by suggesting the most effective predicate values first.

## Introduction

Artificial intelligence planning has proved useful to solve different problems in robotics and computer science. Planning systems were traditionally handled by experts in the field, but this trend is now changing as technology evolves and gets closer to lay users. Therefore, as robots and complex decision-making systems enter our homes, a need for communication and explanation of the reasons behind the system’s decisions arises.

Suggestions are an example of this kind of communication. A non-expert user may not know all the possible configurations the system may have. Hence, the system itself may suggest potential configurations that could improve its performance, taking into account the configuration values that have already been set (user-provided).

Such suggestions can also be used for explanation purposes. In this case, the system could use suggestions that improve the performance to explain why the performance of the system was not good. Or it can use such elements to show why a specific configuration is better than a different one, which would perform worse.

In this paper, we analyze the case of providing suggestions for predicates in planning domains. A predicate is an assertion that may be true or false depending on the values of the variables that occur in it. In a planning problem, predicates are used to describe the domain, and the values assigned to them define the state at any given moment. This state can then be modified by applying actions, which have effects that modify the values of these predicates of the state. We define suggestions as predicate assignments that improve the plan’s reward, such as preferences over the task execution. As an example, such predicates can be the desired speed in a robotics task. We propose two algorithms capable of providing suggestions. The first one finds out values for unassigned predicates that produce better plans; the second one proposes reasonable changes to already assigned predicates by suggesting values close to the current ones. To do so, our algorithms process a portion of the Space of Plans in search of the best assignment of values to predicates. A Space of Plans is a set of possible plans that bring the system from a start state to a goal state. This subset of the Space of Plans is expressed as a Space of Plans Tree structure that provides a compact representation very convenient for searching and traversing. Then, we demonstrate the ability of the proposed methods to improve the reward obtained by the planner, even when low-performance configurations are initially provided. The methods are evaluated in three simulated robotic-assistive tasks, namely shoe-fitting, user feeding and assisted dressing, where the suggestions relate to user preferences that the planner uses to guide the search. To perform an extensive evaluation, user-specified preference values have also been simulated.

These kinds of robot-assistive tasks are particularly complex as they usually involve close contact with human users. Therefore, there is an inherent danger for the user, and planning the task beforehand permits coping with unexpected behaviors (Canal et al. 2018). Allowing the robot to modify its behavior to adapt to the user can improve the performance of the task when assisting a user (Gao et al. 2015; Andriella et al. 2020; Rossi et al. 2017). Preferences have been used to modify the robot behavior through planning (Canal et al. 2019a), and their usefulness has been evaluated by Canal et al. (2021). However, the acquisition of user preferences has proven difficult. The methods we propose in this paper can amend this shortcoming by initializing the user model based on a few preferences and then improving this model by employing the suggested values.

The paper is organized as follows. Section 2 reviews related work in the field, and Sect. 3 analyzes further the motivation behind this work. In Sect. 4 the proposed algorithms are described, while Sect. 5 evaluates their performance. Finally, Sect. 6 provides conclusions of this work and envisaged further research.

## Related work

Our proposed work is closely related to and inspired by different topics. We build on top of the concept of planning “excuses” (Göbelbecker et al. 2010), which are defined as the changes needed in the state to find a solution when no plan could be found. This concept was explored by Martínez et al. (2017) to guide a human teacher when the plan could not be solved. These excuses were also used to find alternative models to explain unexpected states. Similarly, we seek the predicates that can improve the planning performance and provide them as suggestions to the user. We do so by finding the differences between planning states that may cause the largest differences in the plan’s reward.

Therefore, our proposed methods are related to the concept of human-aware task planning and human-in-the-loop planning, where the planner takes into account both the human and the robot’s actions and abilities to improve task performance. Alami et al. (2006) proposed a scheme to integrate humans in the robot control architecture. In it, the abilities and constraints from the users, their needs and their preferences are taken into account in the planning process. The preferences are defined over states, resulting in some states being preferred to others, or as preferred and disliked action sequences along with costs. The authors do not specify how are such preferences represented. In contrast, our approach specifies concrete preference values that are used by the planner to adapt the action selection to them based on the expected produced reward. Cirillo et al. (2010) proposed a planner able to take into account forecasted human actions that constrain the planner allocation of tasks to the robot, but also create new robot goals. Other examples include the Hierarchical Agent-based Task Planner (HATP) (De Silva et al. 2015), where agents are taken into account as first-class entities and user-defined social rules describe the acceptable behaviors of the agents, allowing the creation of plans that take the user safety and comfort into consideration. Fiore et al. (2016) presented a system designed to consider human preferences in human–robot collaboration tasks. In it, the robot can assume different roles and plan the actions for the human, to which it suggests which actions to perform, and also acts as a human assistant. The system can compute a plan for the robot while taking the human into account, but preferences and adaptation are not considered. Chakraborti et al. (2018) show how to project robot intentions during plan executions to assist human–robot interactions using an augmented reality system. The proposed system can reduce the ambiguity over possible plans during task execution and plan generation. In this system, the robot can combine the plan cost with the ability to reveal intentions to improve interaction and task performance. Their goal is to reduce ambiguity over a plan. In contrast, we aim to provide suggestions of preference values that would improve plan performance. The works above mainly assume that the values of the user preferences, if present, are known. Contrarily, in this work we use the concept of suggestions to assess how the task performance could be improved when there are unassigned predicates, such as preferences, which may be unknown. Users are fully modeled by Umbrico et al. (2020), where a user profile is built into an ontology that includes semantics for representing the physical and cognitive capabilities of a person. The proposed system is able to adapt the robot behavior to the user profile in socially assistive robotics scenarios. This work describes a wide framework composed of several modules including planning for action selection based on user need. Conversely, our proposal focuses on the planning part and deals with specific tasks where the preferences help to select how is the task performed, and in discovering what preference values improve the task’s performance.

Our algorithms are based on analyzing the Space of Plans in search of general predicate suggestions, that is, predicates that are missing but that knowing them would help produce better plans. In this paper, we use the example of preference predicates in assistive scenarios. This could also be seen as a preference elicitation process, where we obtain preference suggestions based on already known values. Das et al. (2019) propose a method for eliciting preferences from a human expert while planning. Their approach uses hierarchical task network (HTN) planning to identify when and where the expert guidance will be useful and seek expert preferences to improve the planner decisions. In our case, we similarly suggest user preferences based on the planner reward function without the need of recurring to the human expert during the planning process. However, in our approach, the expert is the one generating the reward function that uses the preferences. Similar to our approach, Kim et al. (2018) perform a search in the space of possible plans to learn to infer final plans in human team planning. They the cost of plan candidates as the reinforcement learning signal, while we perform a search on the space of plans itself to find suggestions to planning predicate values that improve plan’s reward. Another example of the use of plan trees is the one by Shmaryahu et al. (2016), where those are employed for network penetration testing.

The use of preferences to guide search has been investigated by other authors too. Domshlak et al. (2011) review the use of preferences in AI. In the case of planning, PDDL3 (Gerevini and Long 2005) explicitly integrated preferences in the language. They are represented as conditions that do not need to be true to achieve a goal or precondition, but achieving them is desirable. In contrast, we do not use preferences as conditions but we see them as predicates that guide the search by modifying the associated costs and rewards, allowing the use of general purpose planners. Baier and McIlraith review preference-based planning (PBP) in Baier and McIlraith (2008), where preferences are used to distinguish plans by quality and argue for the need for reasoning over preferences when generating a plan, obtaining the most preferred plan. In this case, preferences are specified by an ordering function over the space of plans, where some plans are preferred to others. In our case, preferences are explicit and modify the plan, representing preferences over the task rather than on the plan. We do not use the concept of preferred plan, but the one of preferences that affect the resulting plan. Bidoux et al. (2019) use the PDDL3 language to model preferences over the plans, like in PBP. They use multi-attribute utility criteria (MAUT) to then plan with these preferences using a multiple criteria decision analysis (MCDA). More examples of uses of preferences include another method proposed by Sohrabi et al. (2011), which generates preferred explanations for the observed behavior of the system using planning. A survey on preference-based reinforcement learning by Wirth et al. (2017) reviews works using preference-based reward functions obtained from experts. Monte Carlo Tree Search algorithm using preferences to guide search can be found in Joppen et al. (2018). In this case, an ordinal MDP is solved where the reward function is defined on a qualitative scale, where states can only be compared preference-wise (i.e., one state is preferred to another). Therefore, here the preferences are over states and allow them to be compared, similarly to the concept of preferences in PBP. Another reinforcement learning algorithm that benefits from the use of preferences is presented by Pinsler et al. (2018). Their method learns the reward functions from the robot and human perspectives (user preferences). Preference feedback is used instead of absolute feedback, where preferences are stated over the space of outcomes. Besides planning, preferences have been also used for different applications such as the meeting scheduling problem. An example of this problem can be found in BenHassine and Ho (2007), where a multi-agent approach is used to solve the meeting scheduling with conflicting preferences by allowing the relaxation of some of these preferences. Preferences are represented as soft constraints, in the form of weights reporting the degree of preference of having a meeting on a specific date. Preferences are also used to guide search in BDI agents by Visser et al. (2016). In this case, the preferences are specified in terms of properties of goals and resource usage, allowing users to choose between different plans and determine the order in which to pursue different goals. Preferences specify values that must appear in the plan. In the case of this work, preferences affect the types of actions that are selected by the planner, but the planner does not compute the values of such variables. Behnke et al. (2017) present a mixed-initiative planning approach where the interaction between the user and the planner as a process to determine user preferences toward the plan. The proposed idea is to use the interaction to elicit the preferences of the user *while* planning. In our case, we intend to find the best values to those user preferences through exploring the space of plans. However, using preference values may also be seen as mixed-initiative planning where the human input is used by the planner to compute the best plan. In robotics, (Jiang and Arkin 2015) present mixed-initiative human–robot interaction (MI-HRI) as a collaboration strategy between humans and robots. In it, they achieve a common goal by exploiting their complementary capabilities. Our approach does not actively use the human but instead relies on offline data of computed plans to find the best suggestions to preferences. Similar to our idea of suggestions, (Chun et al. 2003) propose a method for preference estimation. In them, they find optimal solutions to the meeting scheduling problem with unknown preferences. Their method works with analyzing responses given during negotiation to estimate the preference values, which are values that must appear in the decision outcome, such as meeting times. Our proposal also looks into the estimation of preference values, but focusing on the impact of the preferences in the plan performance.

Finally, we have also found inspiration from the Explainable AI (XAI) and Explainable AI Planning (XAIP) communities. In XAIP (Fox et al. 2017), the goal is to present the user with explanatory answers to questions regarding action selection, action alternation, efficiency or affordability of the proposed plans. One way of answering such questions is by proposing alternative plans, by replanning from a user-provided state. Some works have tackled the explainability problem by analyzing the space of possible plans, such as Eifler et al. (2019) who look into explaining the space of possible plans by using plan properties. These properties are Boolean functions that capture the aspects of the plan the user cares about. This concept is closely related to that of user preferences, and we find possible extensions of the proposed work to XAIP. Our Space of Plans representation may be used to analyze the space of possible plans and provide for quick comparisons between plans (known as contrastive explanations). Our methods look into differences between plans to find those with maximal reward, but this could also be applied to compare plans and find the differences that explain the action selection to the user. We believe our algorithms can help to provide information on why some plans may have a better performance than others, thus contributing to plan explanations and avoiding failures.

Even though there has been a lot of research involving preferences, we believe our proposed method is novel in the use of suggestions for improving task performance for planning and decision making, and the use of preferences is a good example of it.

## Motivation

There are not many examples in classical planning where the initial state can be modified at will before starting the task. In classical planning, the planner tries to modify the state using the available actions and operators. However, some elements of the task may be modified when facing the real world. A clear example of it may be that of robotics and, more specifically, collaborative and assistive robotics where humans take part in the planning process.

In such a context where human help can be used, the system or robot can benefit from human interactions and provide information relevant to the task. Therefore, given a state that is not ideal, it can *suggest* changes or additions to the initial state that may lead to a better performance in the task. These suggestions could be obtained either by questioning the user, asking for a change, or just guessing the state of some unknown predicate, knowing that such information may improve the execution performance.

A clear example, which we will use to illustrate the methods of the paper, would be one of preference and user limitations in an assistive robotics task.

### Planning with preferences and limitations

First, we want to ground the definition of preference in the case of our planning domains. Preferences in planning can be defined as soft goals and conditions as in PDDL3 (Gerevini and Long 2005), or can be related to plan ordering (Baier and McIlraith 2008).

#### Definition 1

A task planning problem $\Pi = \langle S, A, T, s_0, g \rangle$ is defined by the set of discrete states *S*, the set *A* of actions that modify the state, the state transition function $T : S \times A \rightarrow S$, the initial state $s_0 \in S$ and the goal state $g \in S$. A solution to this problem is an action sequence starting at $s_0$ that modifies the state using the actions in *A* to achieve the goal state *g*. Each state $s \in S$ is a subset of a set of predicates *P*, and each action $a_i \in A; a_i = \langle p_{a_i},e_{a_i}\rangle$ is a pair composed of the preconditions $p_{a_i}$, the predicates that must be true for the action to be applicable, and the effects $e_{a_i}$, the predicates that reflect the state changes produced by the execution of the action.

State predicates not appearing in the effects of an action remain unchanged by the application of that action. This is known as the STRIPS assumption to avoid the representational frame problem.

For more generality, we will denote the preference predicates as *suggestible* predicates. We define a suggestible predicate or preference as a predicate that is assigned a certain value, appears along with a certain action in the plan and produces some reward when it is present in the state. Such suggestible predicates do not affect the possibility to reach the goal but affect how the goal is reached and which actions are selected. They are used to guide the search and, instead of being conditions that must hold or identifying a plan as most preferred, they are predicates that may or may not hold and as a consequence produce different rewards or costs. Our notion of preferences is the one defined in Canal et al. (2017), where the preferences are used either to guide the action selection process or to modify how a specific action is executed (as a parameter to the action). We will use the example of the robot’s movement *speed* preference, which may take the values of quick, slow or medium. Those three values are the domain of the predicate. The planning domain may have some reward or penalization when using some specific actions. For instance, using a quick movement action when the preference is set to slow would penalize the final reward. However, the planner may still choose that action to complete the task, and the predicate is not required by any of the actions. Thus, *speed* is a suggestible predicate. Other preferences may include but are not limited to robot proxemics and verbosity. Appendix A1 shows further examples of the link between preferences, actions, and the obtained reward.

#### Definition 2

A suggestible predicate $p \in P$ is a predicate such that there is no action $a_i \in A$ in which $p \in e_{a_i}$ or $p \in p_{a_i}$ and $p \notin g$, but it can be that *p* appears in *R*, where *R* is a reward or metric function to be maximized.

The definition of preferences as suggestible predicates allows them to be used by the planner to guide the search, without constraining or forcing the planner to choose those actions that comply with the preferences. This also allows the use of almost any available planner, as preferences are not represented as soft constraints. This means that the planner can use actions that may help achieve the goal even when those do not satisfy the preferences, for instance in cases where those that satisfy them may not be applicable or helpful to reach the goal.

## Providing suggestions

In this section, we propose the Space of Plans Suggestions (SoPS) algorithm to provide suggestions to a set of predicates $Q \subseteq S$.

### Definition 3

A suggestion $q = \{(p, v)\ |\ p \in Q, v \in Domain(p)\}$ is a set of value assignments to predicates such that the reward increases when planning using them.

In our example, a suggestion may be $q = \{(speed,\ quick)\}$.

### Definition 4

A Space of Plans is a set of valid action sequences that bring the system from an initial state $s_0$ to a goal state *g*.

The algorithm analyses a subset of the Space of Plans to provide the suggestions. Its goal is to determine which predicates have more impact on the reward, to suggest those first. Therefore, it needs as an input a subset of the Space of Plans corresponding to the plans obtained by combining the different *suggestible* predicates and obtaining a plan with them, along with their associated plan reward. This subset of the Space of Plans is compiled as a tree for efficient suggestion search. In the case of the *speed* preference, the Space of Plans would include all the plans achievable with all the combinations of *speed* values and the values for the rest of suggestible predicates.

### The space of plans tree

We compile the subset of the Space of Plans into a tree data structure where each branch is a complete plan, similar to the policy trees used in contingent planning (Hoffmann and Brafman 2005). Therefore, all the plans with a common prefix or starting sequence of actions begin at the root node and branch when the plans differ. Accordingly, all the leaves of the tree are actions that produce a goal state.

**Fig. 1:**
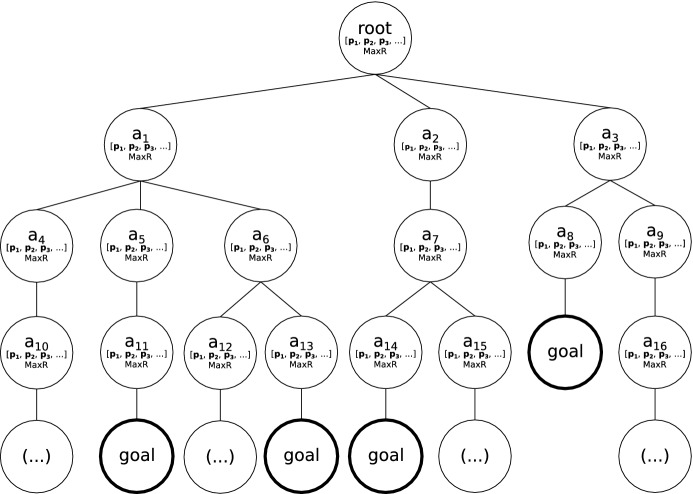
Example of a Space of Plans Tree. Each node $a_i$ represents an action of the tree. Nodes labeled as goal are leaves whose branch is a complete plan to the goal

Each node of the tree keeps a list with the set of suggestible predicates that produced the plan, along with the plan’s reward. This information is kept at each node for all the plans that reach the node. Moreover, the maximum reachable reward is kept at each node for efficient retrieval from the node’s branch. This ensures by construction that the reward of all the children nodes is taken into account. An example of a Space of Plans Tree is shown in Fig. 1. As it can be observed, each node stores the matrix of predicates for all the plans that go through it, and the index to the maximum reward child. For instance, for the $a_1$ node, $maxR_{a_1} = \max ({maxR_{a_4}, maxR_{a_5}, maxR_{a_6}})$.

This representation provides a compact and efficient data structure on which we can perform the search.

In order to populate the tree, the subset of the Space of Plans is generated by executing the planner with changing conditions in the problem file. Therefore, for all the combinations of suggestible predicates, we generate one or more plans (depending on whether stochastic planners are being used). In the *speed* predicate example, we would obtain a plan setting the predicate to all of its possible values (quick, slow, and medium), along with the combinations for the rest of the suggestible predicates. Then, the list of plans is traversed to build the tree, adding new action nodes when new branches are found. When the action node already exists in the tree, the suggestible predicates of the plan along with their associated rewards are added to the node. The computation subset of the Space of Plans may be computationally expensive and is computed offline as a preprocessing step. We only consider a subset of the whole space due to ease make its computation more feasible.

### Max-reward traversal


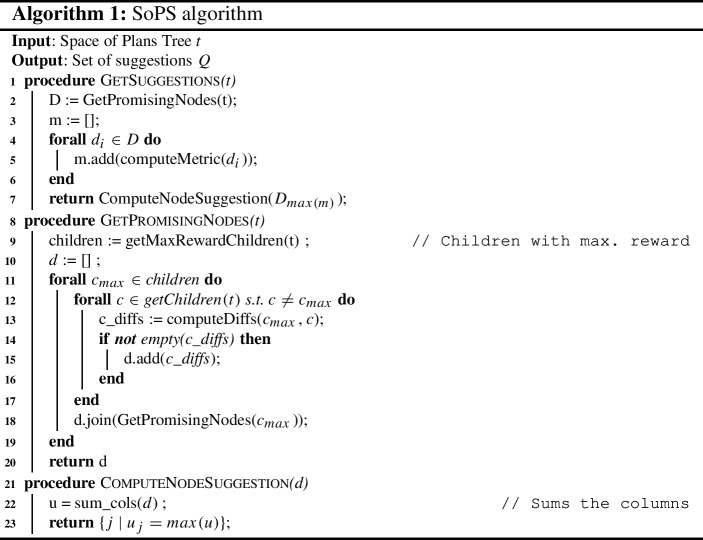


The SoPS algorithm (see Algorithm 1) performs a maximum reward traversal of the Space of Plans Tree to obtain a set of suggestions to unknown suggestible predicates that improve the plan’s reward. For this reason, the already known predicates belonging to the suggestible set *Q* are fixed along the tree. To this end, all the branches belonging to plans generated with predicates whose value is different to the fixed one are pruned, and their rewards are discarded, keeping only the branches belonging to unknown predicates.

To start, the algorithm searches for the promising nodes in the tree (see the GetPromisingNodes procedure). A promising node is a node of the tree such that it is a child with a maximum reachable reward.

#### Definition 5

A promising node *m* is a node in the Space of Plans Tree such that $m \in C_n$ and $\not \exists a_i \in C_n, a_i.MaxR \ge m.MaxR$, where $C_n$ is the set of children of the node *n* and $a_i.MaxR$ is the reward associated to a node $a_i$.

For each of those nodes, we compute a Boolean difference matrix $D^n$ (line 13) such that

1 $$\begin{aligned} { D^n_{i,j} = (p_{j,m} \ne p_{j,i})\ \forall i \in C_n\setminus \{m\}, j \in Q,} \end{aligned}$$

where *m* denotes the child with maximum achievable reward, *Q* is the set of suggestible predicates. With $D^n$ we can compute a set of candidate suggestions for each node *n* (see GetSuggestions procedure). To do so, we flatten the matrix into a vector *d* where $d_j^n = \sum _i{D^n_{i,j}}$. With *d*, we can obtain the set of candidate suggestions *u* (see ComputeNodeSuggestion procedure) as

2 $$\begin{aligned} u = \text {arg}\,\max \limits _{j}\,d_j. \end{aligned}$$

Therefore, the candidate suggestions are the predicates belonging to the maximal child whose values are more different in comparison with its siblings. Those are the predicates that have more impact on the difference of reward, and the ones that make this reward maximal.

Along with the candidate suggestion, a significance metric is computed for all the promising nodes (line 5). This metric is an indicator of how different is the maximum reward child of the node in contrast with the other children. We propose the following metric *f*, which computes the average reward difference between the child with maximum reward *m* and the rest:

3 $$\begin{aligned} f(n) = \frac{\sum _{i}{r_{max} - r_i}}{N-1} = r_{max} - \frac{\sum _{{\{i \in C | i \ne m\}}}{r_i}}{N-1}, \end{aligned}$$

where $r_{max}$ is the maximum reward of all the children of the node *n*, and $r_i$ are the other child rewards.

The rationale behind the metric in Eq. (3) is that child plans that have a greater average reward difference are better candidates at showing which suggestible predicates can make more difference. Subsequently, the output suggestions are the candidate suggestions of the node with the highest metric. Note that in case of a tie in Eq. (2), more than one predicate will be suggested. Moreover, along with the predicate that makes the difference, the algorithm provides an assignment to each of the suggested predicates, which are the values assigned to the predicates in the selected node.

The proposed SoPS algorithm (Algorithm 1) can be executed iteratively in order to obtain new suggestions until all the suggestible set has been determined. To do so, the values of the known suggestible predicates^1^ can be fixed beforehand. More specifically, the algorithm goes over the tree pruning the branches or discarding those that do not satisfy the fixed predicates. The fixed predicates are then also taken into account in Eq. (1), where the fixed predicates are ignored in the computation of the differences matrix.

### Suggesting changes to known predicates

Once we are able to provide suggestions to unknown predicates, we can go a step further and propose *changes* to some of the fixed (already defined) suggestible predicates. This would provide further improvement of the plan performance, at the cost of slightly modifying the user-defined values.

However, the system shall not completely ignore the defined predicates, as they may be given a specific value for a reason. Therefore, we propose to only modify the predicates when the received suggestion’s value is *close* to the defined value. The notion of closeness can be left to the user to be defined. In the case of an ordinal set of values for a predicate, this closeness can just be the arithmetic difference and a defined value of maximum acceptable difference for a change. In the case of the *speed* preference example, we could define the distance between quick and medium, and between medium and slow to be one unit, and the distance from slow to quick to be two units. Thus, the quick value would be closer to the medium value than to the slow one.

#### Definition 6

A change *c* is a suggestion such that $c = \{(p, v)\ |\ p \in Q, v,v' \in Domain(p), (p,v') \in D, sim(v, v') \le T\}$, where *D* is the set of predefined predicates, *sim* is a similarity function, and *T* a user-defined threshold.


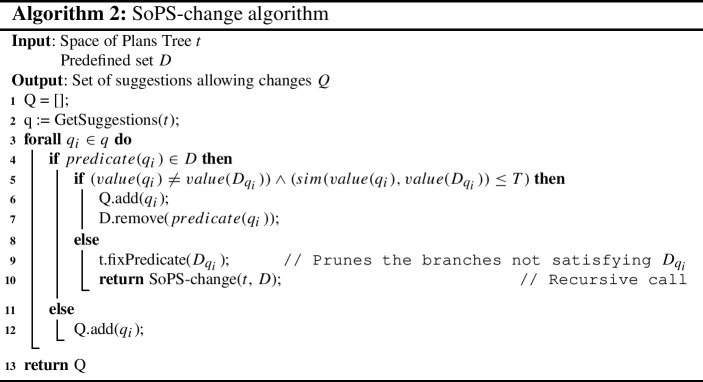


The proposed method for changes is the following. First, we obtain the suggestions from the whole Space of Plans Tree, ignoring the predefined predicates. If the suggestions are new, we add them to the set. Otherwise, the predefined predicate will take the suggested value if the similarity between the values is close enough, as defined above. Algorithm 2 shows the variation of the method including changes.

## Experimental evaluation

To evaluate the proposed algorithms, we have designed three domains in which preferences can play an important role in the decision process and modify the plan to be executed. We have focused on the evaluation of the proposed algorithm given a preference-based reward function. The domains relate to assistive robotics scenarios, consisting of assistive feeding, shoe-fitting and assisted jacket-dressing.

The domains have been written in the RDDL language (Sanner 2010). This language allows for richer reward function definitions, suitable for the integration of suggestible predicates as defined above. See Appendix A1 for more details on the reward function definition. As a plan solver, we have used PROST (Keller and Eyerich 2012) to compute the Space of Plans and to execute all the experiments^2^. The experiments have been run using the ROSPlan framework with the RDDL extension (Canal et al. 2019b).

### Definition of the domains and preferences

Next, we describe the implemented domains and preference options. The domains have been defined such that there are many equivalent actions and different paths to the same goal. For instance, there may be interchangeable actions that produce the same effects but have different executions. Some preferences are used to aid the selection of these alternative actions, while others such as the speed are used as an action parameter. The preferences and suggestible predicates define the final obtained reward and thus guide the planner toward choosing the actions that comply with them. Although out of the scope of this paper, we show in the supplementary videos^3^ the implementation in a real robot of some of the actions corresponding to the different preferences in the three domains. A user study using the domains described in this paper that evaluates the usefulness of the proposed preference model and the ability of the users to recognize the preferences can be found in (Canal et al. 2021).

#### Feeding task

The feeding task completes when the user has been fed (at least *N* spoonfuls completed). The actions available in this domain are listed in Appendix A2.

The preferences involved in this task are head mobility, head proxemics (closeness of the robot to the user), movement speed, applied force, feeding cadence, and robot verbosity.

#### Shoe-fitting task

The shoe-fitting task is completed when both feet have a fitted shoe and the robot’s gripper is empty. The actions in the shoe-fitting domain are described in Appendix A3.

The preferences involved in this task are foot mobility (for each foot), leg mobility (for each leg), speed, applied force, verbosity and requests (defines whether requests to the user should be done or not).

#### Jacket-dressing task

The jacket-dressing task is completed when both sleeves have been fitted until the shoulder and the robot’s grippers are empty. Appendix A4 lists all the actions available in this domain.

The preferences involved in this task are arm mobility (for each arm), speed, applied force, verbosity, and the torso proxemics (how close should the robot get to the user’s torso).

### Effect of the SoPS algorithm

To demonstrate the effectiveness of the SoPS algorithm (Algorithm 1), we have compared the obtained suggestions against random preference value assignments, which represent user-provided values. Note that in a real robot scenario the user would provide these preference values. However, in the experiment of this section, these user-provided (fixed) values will be obtained from previous executions of the algorithm or set at random. To do so, we run the algorithm with random sets of fixed preference predicates, starting with sets of size 0 (without any known predicates) and adding one predicate each time until all the suggestible predicates are known. Thus, the algorithm returns suggestions to the yet unassigned suggestible predicates. Each obtained suggestion is then fixed (and assumed known), and new suggestions are further requested until all the suggestible predicates have been assigned. In this case, the predicates known beforehand (the random sample) were fixed in the Space of Plans Tree and the affected branches were pruned out. Therefore, those predicates are not taken into account by the algorithm.

For each step (new suggestion obtained), we have created 50 sets of random samples of predicates. Afterward, the SoPS algorithm was used to obtain suggestions for the unknown predicates, getting one suggestion at each step until the total number of suggestible predicates was reached.

After computing each suggestion, the planner was used to obtain a new plan, and its final reward was stored. Given that the PROST planner uses stochastic methods and its solution is not deterministic, each plan was computed 20 times. This value was experimentally defined as the number of executions that provided a sufficient number of plans to capture the variability of the obtainable plans for each configuration of the presented scenarios. The results shown in this section are the average of all the 1000 executions (including both the 20 repeated planning attempts and the 50 random samples).

**Fig. 2:**
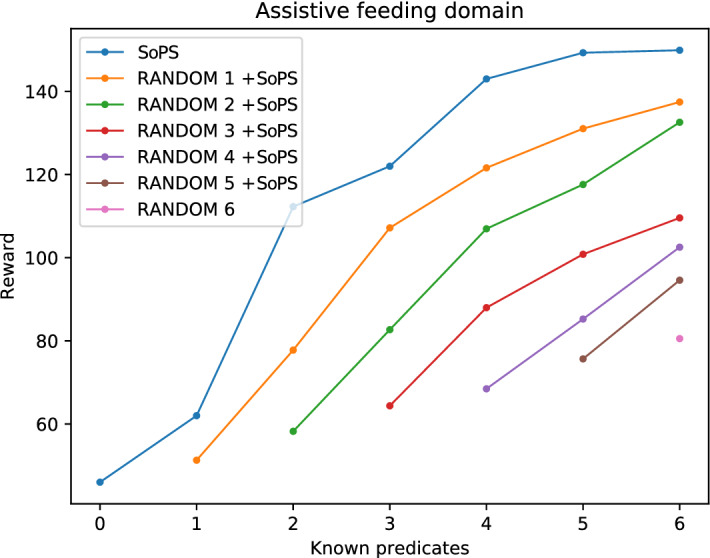
Results with different setups for the feeding domain. Observe how the suggestions provide better rewards in all the cases, even when the system starts with random fixed values for the suggestible predicates

Figure 2 shows the results obtained using the explained procedure for the feeding domain. In this domain, we consider 6 possible preferences or predicates (detailed in Sect. 5.1.1). The SoPS line (in blue) shows the mean reward that can be obtained when no preferences are known (0 known predicates), and the reward that can be obtained when the most promising preferences are determined (up to 6). Note that the videos introduced above show several examples of robot execution with different partial assignments of the preferences.

As it can be observed, the use of the SoPS algorithm highly improves the obtainable reward in all the cases. This is because, even when random fixed predicates don’t give much reward, the algorithm finds suggestions for the other predicates that can improve the total reward. The fact that the initial reward (first point in every line) increases as more predicates are known can be explained because having an extra predicate increments the initial reward (as the suggestible predicates provide extra reward). Note that, as 6 predicates are the maximum, SoPS cannot be applied in the “random 6” case and thus only the mean reward is depicted.

**Fig. 3:**
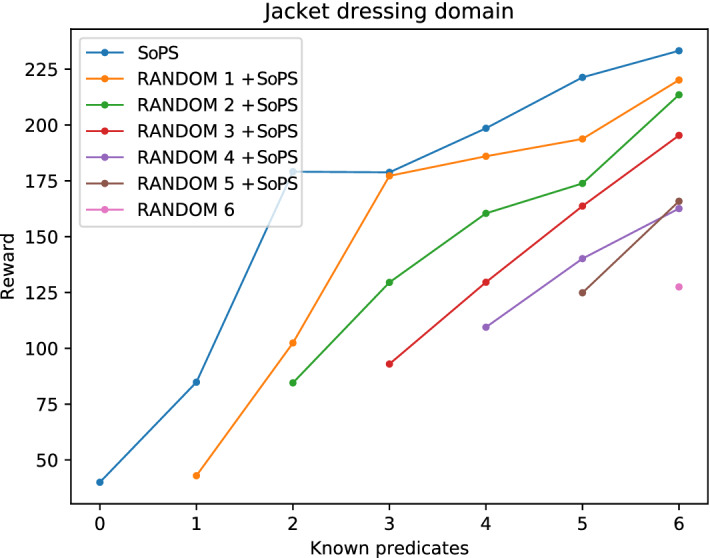
Results with different setups for the jacket-dressing domain. In this case, there is some correlation between some predicates, but the algorithm still produces an improved reward

A similar result is can be seen for the jacket-dressing domain in Fig. 3. Interestingly, a plateau is found between the second and third predicates for the SoPS (blue line). This is a result of two predicates that were suggested together due to being tightly coupled predicates that provide the reward when they are together. In the evaluation, we keep only one of the suggested predicates at each step for easier comparison. Therefore, when obtaining the third predicate the algorithm provides two suggestions. After fixing the first one (predicate 3), the algorithm returns the second suggestion for predicate 4 (which was already suggested in the previous iteration). Therefore, the reward of both predicates is obtained when the second one is suggested.

**Fig. 4:**
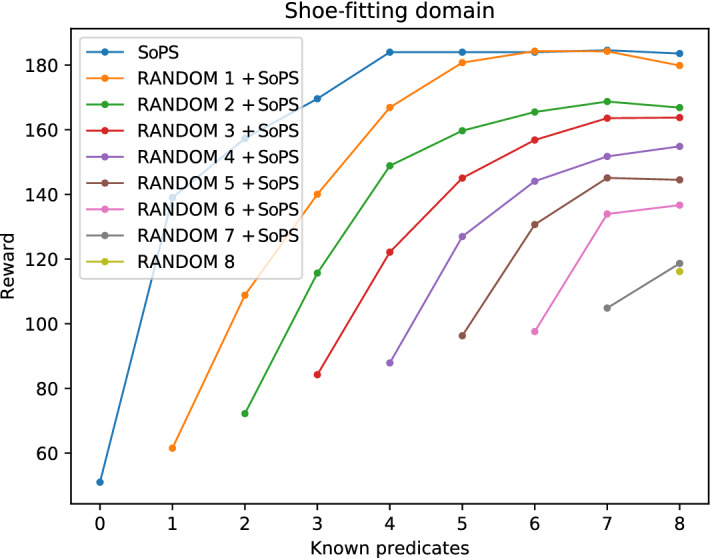
Results with different setups for the shoe-fitting domain. This domain has some predicates that do not improve the reward, and those are suggested at the end when the maximum reward has been obtained

Finally, Fig. 4 shows the same behavior regarding the random pre-assignment of predicates and the algorithm, but plateaus can be observed at the end of the assignments. This is due to the superfluous predicates present in the domain. These superfluous predicates do not increase the reward. Therefore, those are obtained as a suggestion once the useful predicates have been already suggested. A more detailed analysis of the superfluous predicates can be found in Sect. 5.4.

### Improvements by allowing changes with SoPS-change

A new experiment has been performed to analyze the effects of allowing changes in fixed suggestible predicates with the SoPS-change algorithm. These changes are suggestions to already assigned predicates keeping into account the current value. The SoPS-change experiment will be performed, as in the previous section, generating random assignments for the value of the preferences. However, this time changes will be allowed if the suggested predicate was already assigned. In general, any change can be allowed by specifying enough distance as a parameter. Given that our domains include physical scenarios, we believe big changes should not be allowed. Therefore, for this experiment suggested changes are only accepted when the suggestion obtained is at distance one from the assigned value, so bigger changes are not allowed. In case there is a suggested change that cannot be accepted, we fix that preference to the already assigned value and continue with the following suggestions.

**Fig. 5:**
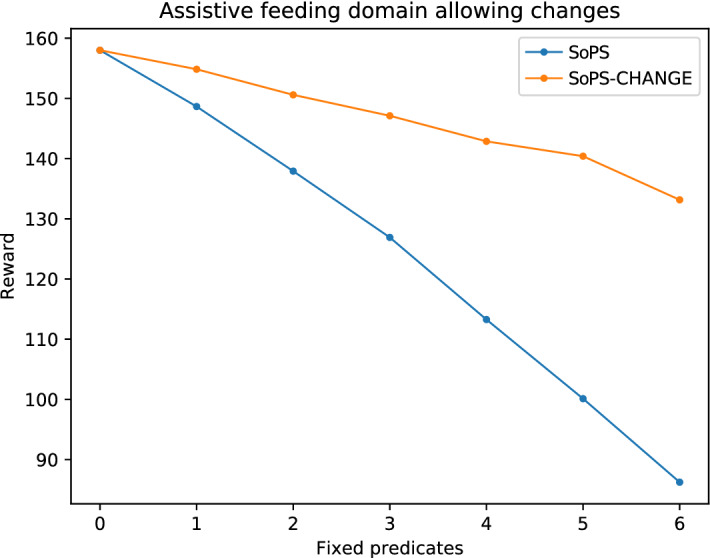
Results for the feeding domain allowing changes. As the number of fixed predicates increases, the reward decreases (as they may not be optimal). Suggesting changes close to the fixed predicates allows to improve the obtained reward

**Fig. 6:**
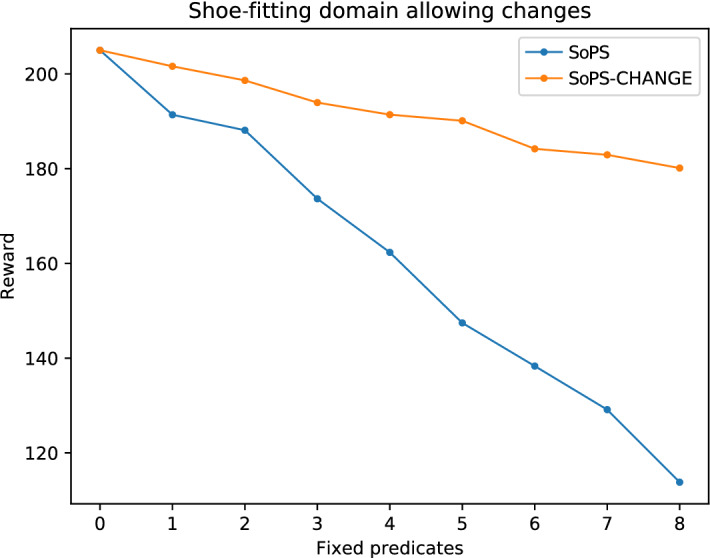
Results for the shoe-fitting domain allowing changes. This domain shows a similar trend, successfully improving the obtained reward with the suggested changes

**Fig. 7:**
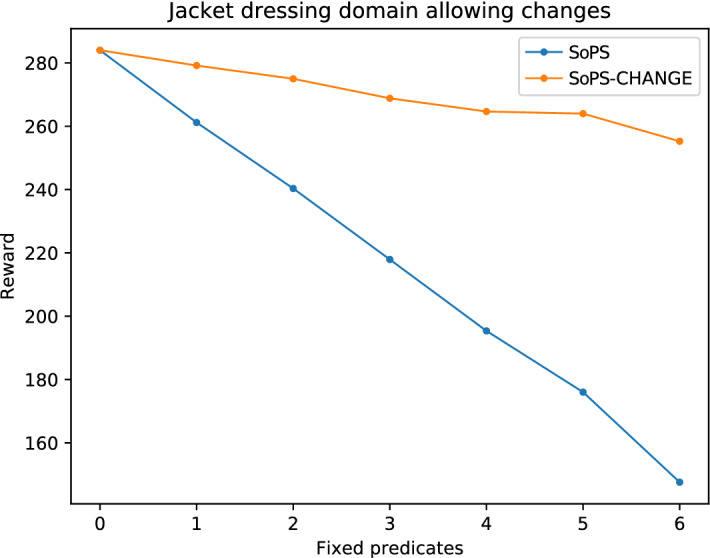
Results for the jacket-dressing domain allowing changes. Change suggestions to the fixed values with a distance of one are enough to improve the final reward

Figure 5 shows the maximum obtainable reward for the feeding case when preference values are fixed, starting with *N* preassigned predicates (horizontal axis) and using the suggested predicates from the algorithms to assign the rest. The figure compares the changes approach to the standard SoPS version. In both cases, the reward values are obtained from the tree, and they represent the maximum obtainable reward as stated in the Space of Plans Tree. Therefore, no new plans are computed this time. Observe that, as in the previous section, the higher the number of random fixed predicates the lower the reward is. But, when changes are allowed, the reward decay is much slower. Notice this happens even in a conservative approach in which only small changes are allowed (so when the suggestion is highly different from the fixed predicate, the suggestion is ignored.) The same behavior can be observed for the other domains, in Figs. 7 and 6.

### Finding superfluous suggestions

The results obtained from the shoe-fitting domain in Fig. 4 show that predicates that do not provide much reward get suggested at the end (as the most promising ones are suggested earlier). To confirm this and analyze its implications, we performed another experiment where more suggestible predicates that do not help to increase the reward were added. We will refer to these suggestible predicates as superfluous predicates.

**Fig. 8:**
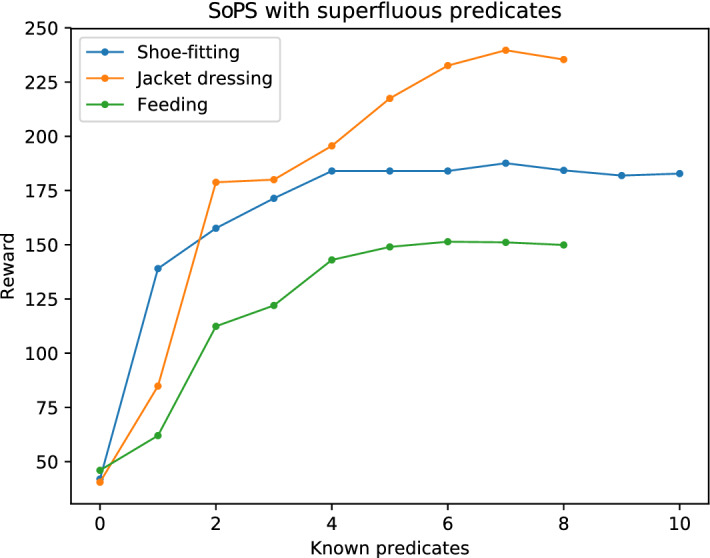
Results with the different domains including superfluous predicates. Our method is able to maximize the reward ignoring the predicates not providing more reward, which are suggested at the end

To this end, we have run the same experimental setup of Sect. 5.2 with slightly modified domains. In them, we added two predicates which are not taken into account in the reward function but allow them to be suggested, being added to the Space of Plans Tree. Later we have executed the SoPS algorithm in them, the results of which are shown in Fig. 8.

As it can be seen, the reward function tends to saturate around the last predicates, while keeping the same shape as in the previous experiment. In the case of shoe-fitting, it is clear that there are many superfluous predicates. The feeding case also shows a third potential superfluous or less-useful predicate, while the jacket-dressing shows that most of the predicates are useful. Slight variations of the tails of the reward plots are due to the stochasticity of the results (which are again an average of all the plan executions).

Consequently, it can be seen that the SoPS algorithm can also be used to determine whether there are superfluous predicates in a domain, which can be used to decrease the size of the search space. However, it can be seen that superfluous predicates cannot usually be detected while obtaining the suggestions, but only when all the suggestible predicates have been obtained. Even so, the computation of all the suggestions is efficient and quick enough to be possible to pre-compute the superfluous predicates beforehand.

## Discussion and conclusions

In this work, we have presented an algorithm to provide suggestions for assigning values to predicates in planning domains. We have defined the concept of suggestible predicates, which are those predicates that help the planner by guiding the search to obtain more reward under some circumstances. Then, we have introduced the SoPS algorithm that uses a Space of Plans Tree built from a pre-computed subset of the Space of Plans. The algorithm traverses the tree to obtain suggestions for predicates such that the final plan’s reward is maximized. A variation of this algorithm (SoPS-change) that suggests changes to already assigned predicates has also been proposed. These changes are considered taking into account the currently assigned value to the predicate.

The algorithms were evaluated in three assistive robotics domains in which the suggestible predicates are preferences of the user that define the robot’s behavior. Our results show that using the values selected by the algorithms improve more the reward in comparison with a random simulation of user selection of the values when computing new plans. The focus of the evaluation was on the ability of the proposed algorithm to provide suggestions to preference values given a preference-based reward function. A more comprehensive evaluation could also be performed by evaluating the preference-based reward function and taking into account feedback from users. This is left as future work.

The proposed methods have their drawbacks. We do not consider the Space of Plans computation as part of the algorithms but as an offline pre-computation step. However, the algorithms need the subset of the Space of Plans as an input, we acknowledge the fact that obtaining such Space of Plans is not computationally cheap. We want to emphasize that we use a subset of it due to the intractability of obtaining the whole space, and the more suggestible predicates the more costly this becomes, which is a limiting factor. This could be partially overcome by starting with a smaller subset of the space and integrating new plans as they are computed during the system’s execution. This would imply that the suggestions might not be the best ones with an incomplete Space of Plans but could be improved over time.

Another limitation is the need for a preference-based reward function. We consider this as an input to the system and created by experts, but the quality of the suggestions will depend on how good is the reward function in terms of consistency between the effect of the preferences and the related actions. If the reward function is not appropriate, the suggestions will still maximize the reward of the system but may not be consistent with what users might expect. Some approaches that adapt the cost or reward function from user interaction to improve the use of preferences (Canal et al. 2019a) could reduce the impact of a poorly defined reward function, but more work can be done in this direction.

The methods proposed in this paper can be used in many other domains apart from those already shown here. We believe the algorithms can also be used to foster plan explainability. For instance, the suggestions provided by our algorithms could also be used to explain to a non-expert user why the planner took an action or another in terms of gained reward, as well as to help the user in selecting the best configuration based on their needs, explaining that assigning a specific value to a predicate can lead to better plans. Although some more work shall be done in this direction, we believe these algorithms can be useful for providing plan explanations, as well as powerful algorithms to analyze the Space of Plans.

## Preferences to guide action selection through the reward function

This section details how the reward function can be defined such that the suggestible predicates help in the action selection process of the planner. For this, we have used the Relational Dynamic Influence Diagram Language (RDDL) (Sanner 2010), which allows the definition of rich reward functions. Such reward function is computed at each time step to provide an immediate value to the metric function that is being optimized. In the case of the reward, the goal of the planner is to maximize its value. Therefore, actions that lead to states providing more reward will be favored. This allows the use of suggestible predicates to appear along with actions in the reward function, such that when the suggestible predicate is present with a specific action, it will provide a positive or negative reward. This will encourage or penalize the use of the action.

In RDDL, we can do this by defining an if-then-else reward function. The function will have a case for each action and related preference value with the form

where *R* is the value that is obtained when the action is executed and the preference_name predicate is present and has the @preference_value.

To ease the description of such predicates in the reward function, we defined a rule-based format that consists of the action, the preference application and the immediate reward value *R*. This is then formatted and added to the domain automatically. As an example, one could define:

which specifies that the action getFood receives a penalization with a value of 15 when either the speed or force preferences are not unknown and they have a different value than the one provided by the preference. Another example of the jacket dressing task is:

which describes that the approachBothArms action is rewarded when the user’s arms mobility is defined as high. This will then be converted to:^4^

Consequently, this process simplifies the definition of the reward function, which can become long and complex. Note that in our approach, we expect an expert in the domain to define the rules that will be compiled into the reward function to successfully lead the planner to choose those actions that receive more reward thanks to the preferences.

## Actions in the feeding task domain

The feeding task domain contains the following actions:

- Get Food: uses the cutlery to get the food.
- Approach straight: approaches the food to the user frontally.
- Approach from below: approaches the food to the user from below (less intrusively).
- Approach from the side: approaches the food to the user sideways, being always visible but not frontally to avoid intimidating the user.
- Feed straight: feeds the user by moving in a straight line and exiting in the same way.
- Feed scooping: feeds the user and performs a scooping action when exiting to ensure emptying of the cutlery.
- Wait for user feeding: waits for the user to get the food.
- Move away: the robot moves back to the starting position.

## Actions in the shoe-fitting task domain

The shoe-fitting task domain includes the following actions:

- Request foot reachable: requests the user to move the foot closer.
- Request foot visible: requests the user to put the foot in the robot’s sight.
- Grasp shoe: it grasps the shoe (from the user, as a handover).
- Approach from top: it approaches the user’s foot from the top.
- Approach right/left: it approaches the user from either side.
- Approach from below: approaches the user from below.
- Insert straight: inserts the shoe in a straight movement, without forcing the ankle.
- Insert curved: shoe insertion forcing a bit the ankle to fit correctly the heel.
- Insert right/left: inserts from the side following the foot’s shape.
- Release simple: releases the shoe and moves away.
- Release push: it pushes the shoe a bit before releasing it to ensure fit.

## Actions in the shoe-fitting task domain

The jacket-dressing task domain consists of the following actions:

- Approach single-arm frontal: approaches a sleeve to a single arm from the front (visible to the user).
- Approach single-arm rear: approaches the sleeve to the arm from behind (more comfortable as less movement is involved).
- Approach arm side: approaches a sleeve from the side (from outside the body to the right/left arm).
- Approach both arms: approaches both sleeves together from behind.
- Insert sleeve from the front: inserts the sleeve in a frontal manner (doing so makes it impossible to insert the other sleeve frontally).
- Insert sleeve straight: inserts a sleeve from the side, with the stretched arm.
- Insert both sleeves: inserts both sleeves together from behind.
- Drag forearm frontal: drags the sleeve in the forearm from the front.
- Drag forearm straight: drags the forearm sideways.
- Drag both forearms: drags both forearms together.
- Drag upper arm: drags an upper arm.
- Drag both upper arms: drags both upper arms together.
- Release: Drags the cloth to the shoulders and releases the garment.

## Footnotes

1 Predicates can be known because they are provided to the algorithm or because they were obtained as suggestions in a previous execution.

2 The source code of the proposed algorithms can be found in https://github.com/gerardcanal/SoPS.

3 Videos can be found in http://www.iri.upc.edu/groups/perception/preferenceSuggestionsExamples.
